# Alternative pre-mRNA processing regulates cell-type specific expression of the *IL4l1 *and *NUP62 *genes

**DOI:** 10.1186/1741-7007-3-16

**Published:** 2005-07-19

**Authors:** Stefan Wiemann, Anja Kolb-Kokocinski, Annemarie Poustka

**Affiliations:** 1 Molecular Genome Analysis, German Cancer Research Center, Im Neuenheimer Feld 580, Heidelberg, 69120, Germany

## Abstract

**Background:**

Given the complexity of higher organisms, the number of genes encoded by their genomes is surprisingly small. Tissue specific regulation of expression and splicing are major factors enhancing the number of the encoded products. Commonly these mechanisms are intragenic and affect only one gene.

**Results:**

Here we provide evidence that the *IL4I1 *gene is specifically transcribed from the apparent promoter of the upstream *NUP62 *gene, and that the first two exons of *NUP62 *are also contained in the novel *IL4I1_2 *variant. While expression of *IL4I1 *driven from its previously described promoter is found mostly in B cells, the expression driven by the *NUP62 *promoter is restricted to cells in testis (Sertoli cells) and in the brain (e.g., Purkinje cells). Since *NUP62 *is itself ubiquitously expressed, the *IL4I1_2 *variant likely derives from cell type specific alternative pre-mRNA processing.

**Conclusion:**

Comparative genomics suggest that the promoter upstream of the *NUP62 *gene originally belonged to the *IL4I1 *gene and was later acquired by *NUP62 *via insertion of a retroposon. Since both genes are apparently essential, the promoter had to serve two genes afterwards. Expression of the *IL4I1 *gene from the "*NUP62*" promoter and the tissue specific involvement of the pre-mRNA processing machinery to regulate expression of two unrelated proteins indicate a novel mechanism of gene regulation.

## Background

Many mechanisms for the alternative use of promoters, exons and polyadenylation signals within genes are known to significantly contribute to the complexity of the transcriptome [1-6]. These variations increase the number of products that can be generated from the currently recognized 20,000 – 30,000 protein-coding genes of the human genome [7]. For example, alternative promoters are used to confer specificity of mRNA expression in time and space [8,9] and of mRNA translation [10]. Often the N-terminal ends of proteins are altered to generate or remove signal sequences for protein localization [11]. Central exons may or may not be present thus changing the peptide sequence and properties [12]. The alternative use of polyA signals also has effects, for instance, on RNA stability [13,14].

The mechanisms described above all have in common the fact that the elements involved are associated only with the gene being transcribed and not with any other gene. The mechanism of trans-splicing, in which elements from more than one gene are involved in the generation of transcripts, is an open matter of discussion, although it appears to be rare and its function is still not well understood [15]. Overlapping genes and transcripts have been described in many species and occur in several varieties [16-18]. However, in vertebrates, few transcripts have been described which join two genes with different reading frames [19]. We have found evidence for sequence overlap of transcripts from two protein coding genes, *NUP62 *and *IL4I1*, where the latter is expressed in a tissue and cell-type specific manner. Both genes are transcribed from the same promoter and share the first two exons. A similar process has been described for *Caenorhabditis elegans *[20], in which mRNAs of two cholinergic proteins are transcribed from one promoter. Until now, this principle did not appear to be conserved in higher eukaryotes. The *NUP62*/*IL4I1 *genes are therefore the first proof that this mechanism is present in vertebrates. However, in contrast to what has been observed in *C. elegans*, the functions of the two proteins encoded by the one promoter are completely unrelated.

The protein encoded by *NUP62 *belongs to the class of nucleoporins (Nups) and is an essential part of the nuclear pore complex [21,22]. Its N terminus is believed to be involved in nucleocytoplasmic transport, while the C-terminal end contains a coiled-coil structure aiding in protein-protein interactions, and may function in anchorage of the protein in the pore complex (Annotation for P37198 in Swiss-Prot [23]). Nup62, like the other Nups, is conserved in the eukaryote kingdom [24,25]. The *NUP62 *gene consists of a single promoter with a CpG island and three transcribed exons. The protein is encoded exclusively by the terminal exon; the first two exons are non-coding. The second exon is prone to alternative splicing and is not contained in about half of the reported cDNAs derived from that gene (e.g., IMAGE:3050260 [26] and DKFZp547L134 [27]). *NUP62 *is ubiquitously expressed, an observation compatible with its essential role in transporting cargo across the nuclear envelope.

*IL4I1 *was initially identified to be exclusively expressed in B lymphoblasts as a gene that was induced by treatment with interleukin 4 (IL-4) [28,29]. Since then, the encoded protein has been identified as a leukocyte specific L-amino acid oxidase (LAAO; [30]) that specifically oxidizes aromatic amino acids. The protein contains an N-terminal signal peptide, which targets the protein to the endoplasmic reticulum and presumably to the lysosomes [30], where it is believed to be involved in antigen processing in B cells [30] and thus act in the immune response. The gene is reported to be transcribed from a single promoter, which appears to restrict expression to cells of the immune system, mostly in B lymphocytes [31]. It consists of eight exons, and the translation start is located in the second exon. The gene is conserved in eutherian mammals (NCBI HomoloGene:22567), but has not been identified in other eukaryotes and in prokaryotes.

We have identified several expressed sequence tags (ESTs) that indicate expression of *IL4I1 *in tissues other than B lymphocytes, namely human and mouse testis and brain. This expression of the *IL4I1 *gene was apparently driven by the same promoter as the upstream *NUP62 *gene. We have verified expression of the *Il4i1_2 *variant in mouse testis and brain, and thus show that the previously reported *NUP62 *promoter also drives expression of a second gene in a cell-type and tissue specific manner. The mRNA consists of sequence from both genes and two joining exons which are not part of either previously reported gene locus. Our findings indicate a new mechanism of gene regulation in which two genes that encode unrelated proteins share the same promoter but yet are still expressed in radically different cellular patterns. This suggests that the nature of the transcripts and proteins encoded by these two genes is controlled by tissue specific regulation of pre-mRNA processing.

## Results

### The exon structure of variant *IL4I1_2 *joins the described *NUP62 *and *IL4I1 *genes

**Figure 1:**
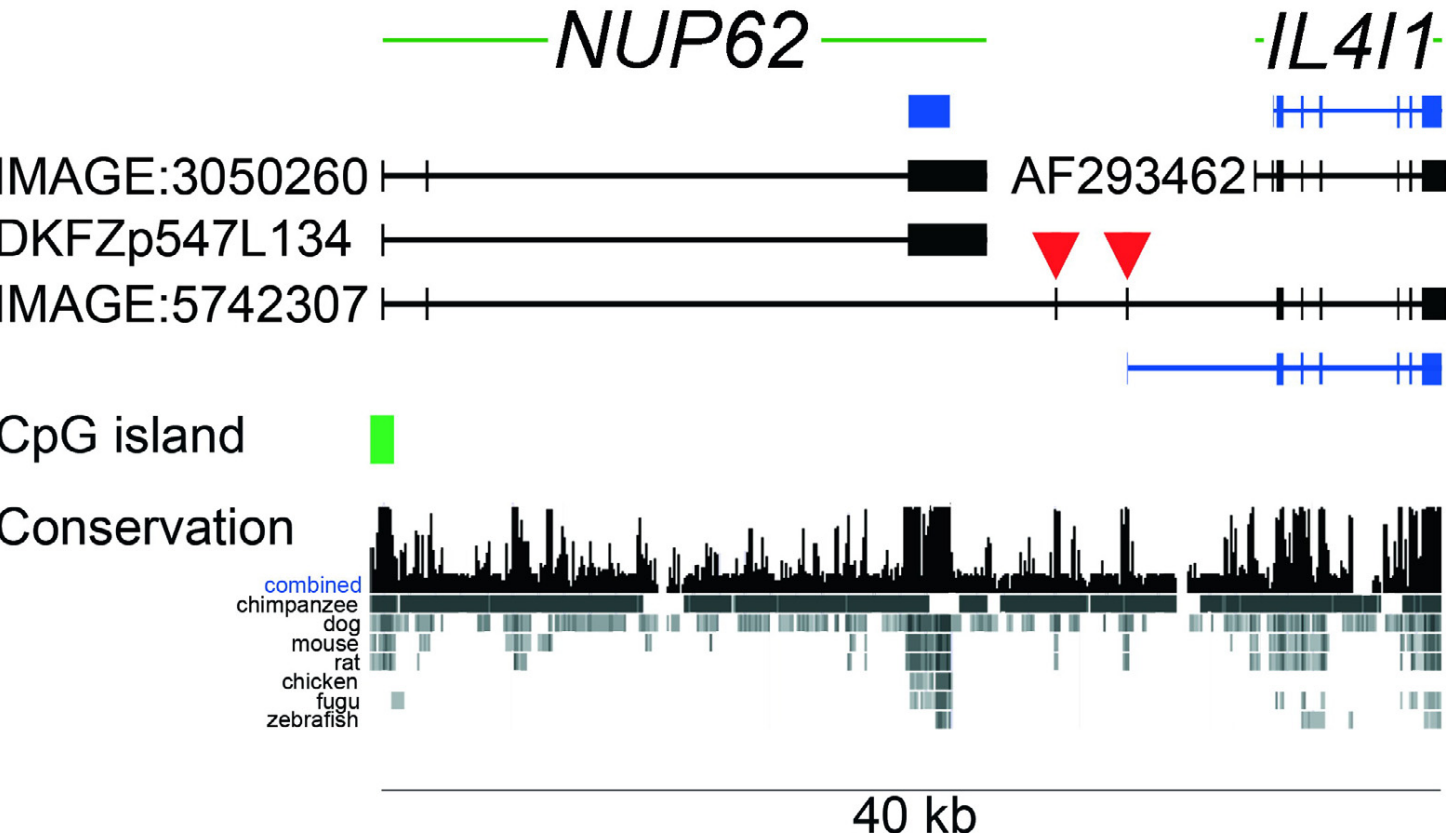
**Structure of the human *NUP62 *and *IL4I1 *genes at chromosomal band 19q13.33**. Genes are both shown from 5' (left) to 3' (right). Exons are represented by vertical bars and boxes, intronic sequence by horizontal lines. The *NUP62 *gene (three exons) is located upstream and tail-to-head to the previously reported *IL4I1 *gene (eight exons). *NUP62 *has two splice variants represented by cDNAs IMAGE:3050260 (includes exon 2), and DKFZp547L134 (skipped exon 2). The Nup62 protein is encoded by exon 3 (blue box). *IL4I1 *does not have reported splice variants and is represented by the sequence AF293462. Translation start of the protein is located in exon 2 (blue bars represent coding region). Several cDNAs were identified to link *NUP62 *and *IL4I1 *transcripts (representative: IMAGE:5742307). The splice form consists of the first two apparent exons of the *NUP62 *gene, two joining exons mapping in between the reported *NUP62 *and *IL4I1 *gene loci (red arrow heads), and exons 3–8 of the known *IL4I1 *gene. Translation start of the protein is in the second joining exon. The sequences of the joining exons are conserved in eutherian species, but not for instance in chicken or fish (taken from UCSC genome browser [54]). The reported promoter of the *NUP62 *gene contains a CpG island, while the promoter of previously known *IL4I1 *does not. The two genes cover 40 kb on the chromosome.

Based on the available sequence information we predicted the gene structure for the human variant *IL4I1_2 *transcript represented by cDNA IMAGE: 5742307 in Fig. 1. To validate this structure we obtained several Mammalian Gene Collection clones that cover the splice variant and sequenced them to completion. One cDNA (IMAGE:4822638; Acc: BC026103) contained two mutations leading to premature in-frame stop codons. A second cDNA (IMAGE:5168029) contained exon 2 (35 nucleotides) of the previously reported *IL4I1 *gene [32], also disrupting the open reading frame (ORF). The remaining clones (IMAGE:5171014, IMAGE:5742307 and IMAGE:4838597) matched the predicted gene structure and thus supported the sequence of the variant. This gene structure includes the presumed first two exons of the *NUP62 *gene which are both part of the 5' untranslated region (UTR). Transcription of that variant is apparently controlled by the promoter that also controls expression of the *NUP62 *mRNA.

The terminal and coding exon from the *NUP62 *gene is not contained in the *IL4I1_2 *variant (Fig. 1). While the initiator ATG of the reported *IL4I1 *ORF is located in exon 2, the first two exons of the known *IL4I1 *gene are absent in the variant. Instead, the variant contains two additional exons (indicated with red arrowheads in Fig. 1) that are located in the region between the previously reported *NUP62 *and *IL4I1 *loci. The latter of the two exons contains the assumed translation initiator ATG.

### The *IL4I1_2 *variant is conserved in eutherian mammals

**Figure 2:**
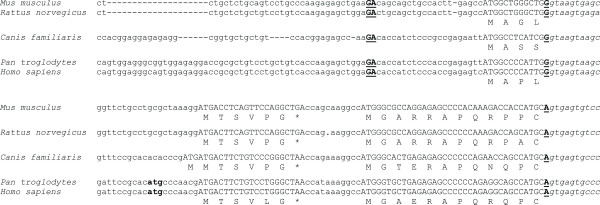
**Alignment of sequences upstream the *IL4I1 *and variant *IL4I1_2 *open reading frames**. Alignments of *IL4I1 *(upper panel) and variant *IL4I1_2 *(lower panel) sequences are shown. The first two exons of *IL4I1 *are shown, and splice sites are underlined and in bold. Ten nucleotides of the next intron are displayed downstream of the first coding exon, which all match the consensus splice donor sequence. Reading frames are in all caps, and the deduced peptide sequences are given below. The human and chimpanzee sequences of *IL4I1_2 *have an upstream and in-frame start codon (bold), which is not conserved in mouse, rat or dog, and which does not match the Kozak consensus rules. All *IL4I1_2 *sequences have a short upstream ORF (all caps) which is conserved in location and sequence. The mouse, rat and human sequences were derived from cDNA sequences, while the chimpanzee and dog sequences were deduced from an alignment of human cDNA with the respective genome sequences [54].

The splice variant is conserved in other eutherian mammals where order and orientation of the *NUP62 *and *IL4I1 *genes are syntenic. Five ESTs from mouse verify the transcription and splicing of the *Il4i1_2 *variant. Like the human ESTs, the mouse ESTs were derived from cDNAs that had been generated from either testis or pooled tissues. One EST was sequenced from rat testis. All these cDNAs contain the first exon of the *Nup62 *gene, two intergenic exons and then exon 3 of the *Il4i1 *gene. There is apparently no homolog of human exon 2 of *NUP62 *in mouse and rat. Mouse *Nup62 *is thus the equivalent of the human splice variant represented by cDNA DKFZp547L134. The location and sequences of the joining exons that are specific for the *IL4I1_2 *variant are conserved between mouse, dog and human. Sequence conservation of the variant joining exons is higher than that of exons 1 and 2 of previously reported *IL4I1 *(Fig. 2). The probable translation initiation codon in exon 4 (exon 3 in mouse and rat) lies within a consensus Kozak sequence context (Fig. 2; [33]). An upstream ATG, which is in frame with the ATG we propose to initiate translation, does not match the Kozak consensus rules. It is present in human and chimpanzee, but not in mouse, rat or dog, and thus is not convincing; we suspect it could be prone to leaky scanning [33]. We conclude that translation either starts at the conserved ATG, or that use of the upstream ATG could possibly change some property of the encoded protein. While the N terminus of Il4I1_2 protein is predicted (SignalP [34]) to be a signal peptide (*P *= 0.969) when starting at the Kozak-ATG, the extended N terminus is predicted to be a signal anchor (*P *= 0.587) and not a signal peptide (*P *= 0.316). An extension at the N terminus might thus change localization of the protein.

All transcripts analyzed had a short six (dog: seven) residue upstream ORF, the localization and sequence of which was conserved. It remains to be determined whether this ORF is expressed in vivo as has been shown for other genes [35]. This ORF is too small and too close to the initiator ATG of the *IL4I1*-ORF to suggest an internal ribosome entry site (IRES) – type mechanism [36].

The *IL4I1 *gene has thus far only been found in eutherian mammals. This is supported by analysis of the genes downstream of the *NUP62 *orthologous genes in non-eutherian species. In *Fugu rubripes*, the next gene downstream of *NUP62 *is a homolog of human integrin alpha 6, and the two genes are oriented tail to tail. In *Gallus gallus*, the next gene downstream is the homolog of a human X-chromosomal gene (FLJ11016) with unknown function, and the genes are oriented head to tail. In *Drosophila melanogaster*, *Nup62 *is followed by a hypothetical WD-repeat protein (CG7989), which is in the opposite orientation (tail to tail) to *Nup62*. The situation in the opossum (*Monodelphis domestica; *thus far the only marsupial species sequenced) is unclear, as the sequence scaffold that covers *NUP62 *terminates 4 kb downstream and no gene is annotated there. However, the two genes that, according to annotation, flank opossum *NUP62 *do not map to the chromosomal region that harbors human *NUP62 *and *IL4I1*. In addition, no ortholog of the *IL4I1 *gene has yet been identified in the opossum genome. Thus, the evidence so far suggests that expression of variant *IL4I1_2 *(just as of original *IL4I1*) might be restricted to eutherian mammals. The sequencing and transcript analysis of more mammalian species will help to uncover the origin of the *IL4I1 *gene and its variant.

### Mature ll4i1 protein and its variant are likely identical in sequence

**Figure 3:**
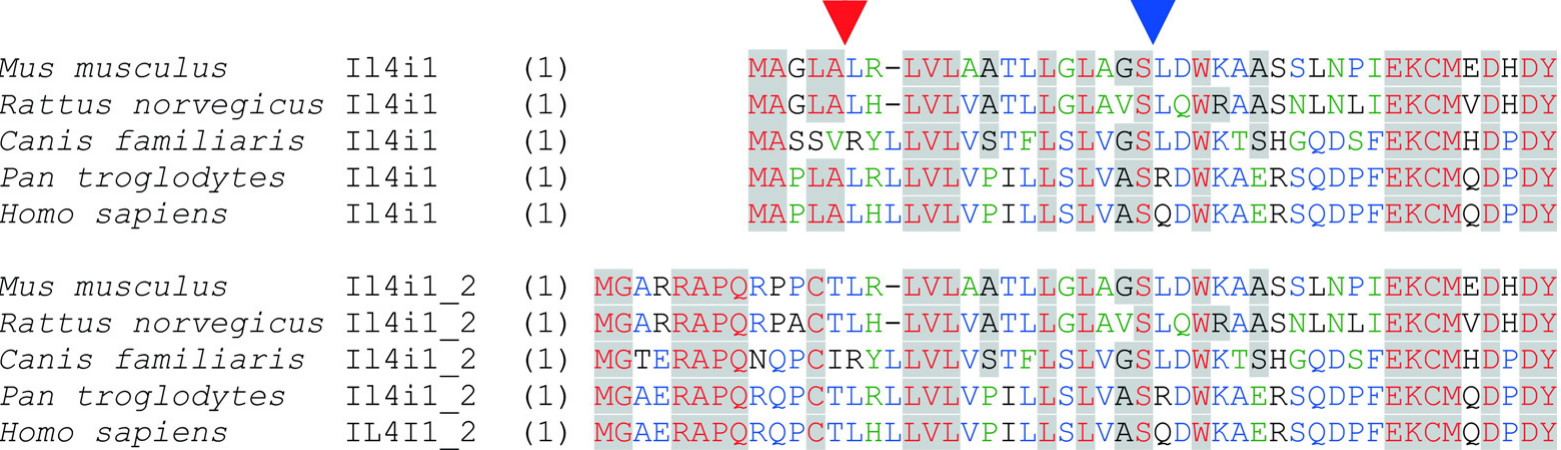
**Sequence alignment of N-terminal ends of peptides encoded by the *IL4I1 *gene (Il4i1) and the novel variant (Il4i1_2)**. Sequences encoded by the first coding exon are different between the two variants, and the splice site is indicated with a red arrowhead. The mouse sequence of Il4I1_2 was derived from the following ESTs: BY100275, BY099330, BY087056, BY092834 and BY088421. The rat sequence was translated from the EST CV117152. Dog and chimpanzee sequences were deduced from aligned genomic sequences [54]. The cleavage site of the signal peptidase is predicted at the same position in all sequences and is indicated with a blue arrow head.

Since the translation start in the previously reported *IL4I1 *transcript differs from that in the variant described here, the two protein products differ at their N termini. Fig. 3 shows a sequence alignment of the N-terminal ends of Il4I1 and the new variant. The N termini of Il4i1 and those of the variant Il4i1_2 are conserved in the species analyzed. The Il4i1 protein has been reported to be transported into the endoplasmic reticulum and the endosomes with help of an N-terminal signal peptide [30]. SignalP predicts such a signal peptide to be cleaved upstream of the glutamine residue at position 22 of the human variant protein. The homologous position is a leucine in the mouse protein and is there predicted to be the cleavage site. The same residue is the cleavage site also in the previously reported Il4i1. Consequently the processed proteins are probably identical in sequence, and only differ in the length of the respective signal sequences. We next analyzed whether the N terminus of the Il4i1_2 variant may serve as signal peptide in vitro and expressed the protein in fusion with green fluorescent protein (GFP) in mammalian cell culture [37,38]. The variant protein was indeed translocated into the endoplasmic reticulum [39] when overexpressed, and had the same localization as an overexpressed Il4I1-GFP fusion protein (not shown).

### The *IL4I1_2 *variant is specifically expressed in testis and brain

**Figure 4:**
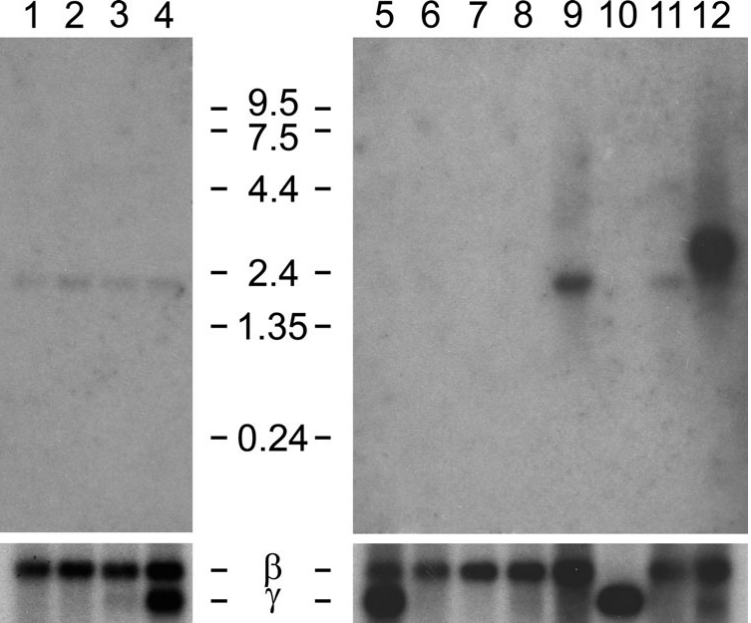
**Northern hybridization with probe specific for the mouse *Il4i1_2 *splice variant**. Blots contained poly A+ RNA from fetal mice 7 dpc (1), 11 dpc (2), 15 dpc (3), 17 dpc (4) and adult mouse tissues (heart (5), brain (6), spleen (7), lung (8), liver (9), skeletal muscle (10), kidney (11), and testis (12). Hybridization was with a probe comprising the joining exons 2 and 3 of the mouse *Il4i1_2 *variant, which are equivalent to human exons 3 and 4 (red arrowheads in Fig. 1). The blots were reprobed for beta actin mRNA as control for RNA content. The probe cross-hybridized with gamma actin mRNA where expressed.

EST evidence indicated that expression of the variant transcript might be tissue specific, as cDNAs exclusively from testis and brain had been sequenced. We analyzed the expression of the variant transcript in Northern blots of fetal and adult mouse (Fig. 4). A probe specific for the variant *IL4I1_2 *was employed, comprising the two joining exons downstream of the *NUP62 *coding exon. These exons are indicated with red triangles in Fig. 1. No expression of the *Il4i1_2 *variant was observed in fetal mice and in most adult tissues. A strong and specific band at 2.45 kb was only observed in the testis. The variant *Il4i1_2 *transcript is predicted to be 2.3 kb in size, not counting the polyA tail, when sequence of the ESTs (Methods) is extended with the known *Il4i1 *sequence towards its 3' end. The human variant *IL4I1_2 *is of similar size. Expression in the brain was expected because of cDNAs and ESTs available from that tissue, but not observed in Northern blot analysis. A smaller RNA of unknown sequence at 1.8 kb was visible in the fetal mouse, and in adult mouse liver, kidney and testis.

**Figure 5:**
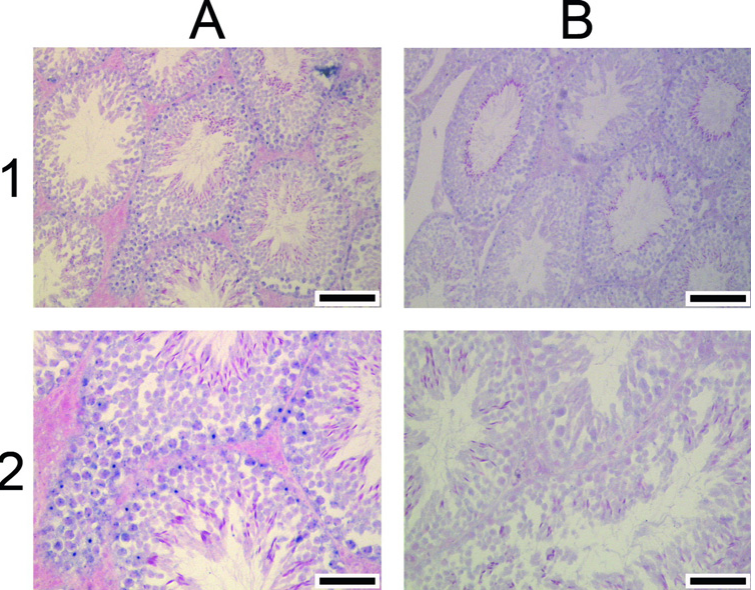
**RNA in situ hybridization of variant *Il4i1_2 *in mouse testis**. Probes were transcribed from the joining exons specific for variant *Il4i1_2*, which are located between the previously known *Nup62 *and *Il4i1 *gene loci. Signals (in blue) obtained with the antisense probe are on the left (A), and those obtained with sense probe are on the right (B). The scale bar is 100 μm in 1A and B, 50 μm in 2A and B.

**Figure 6:**
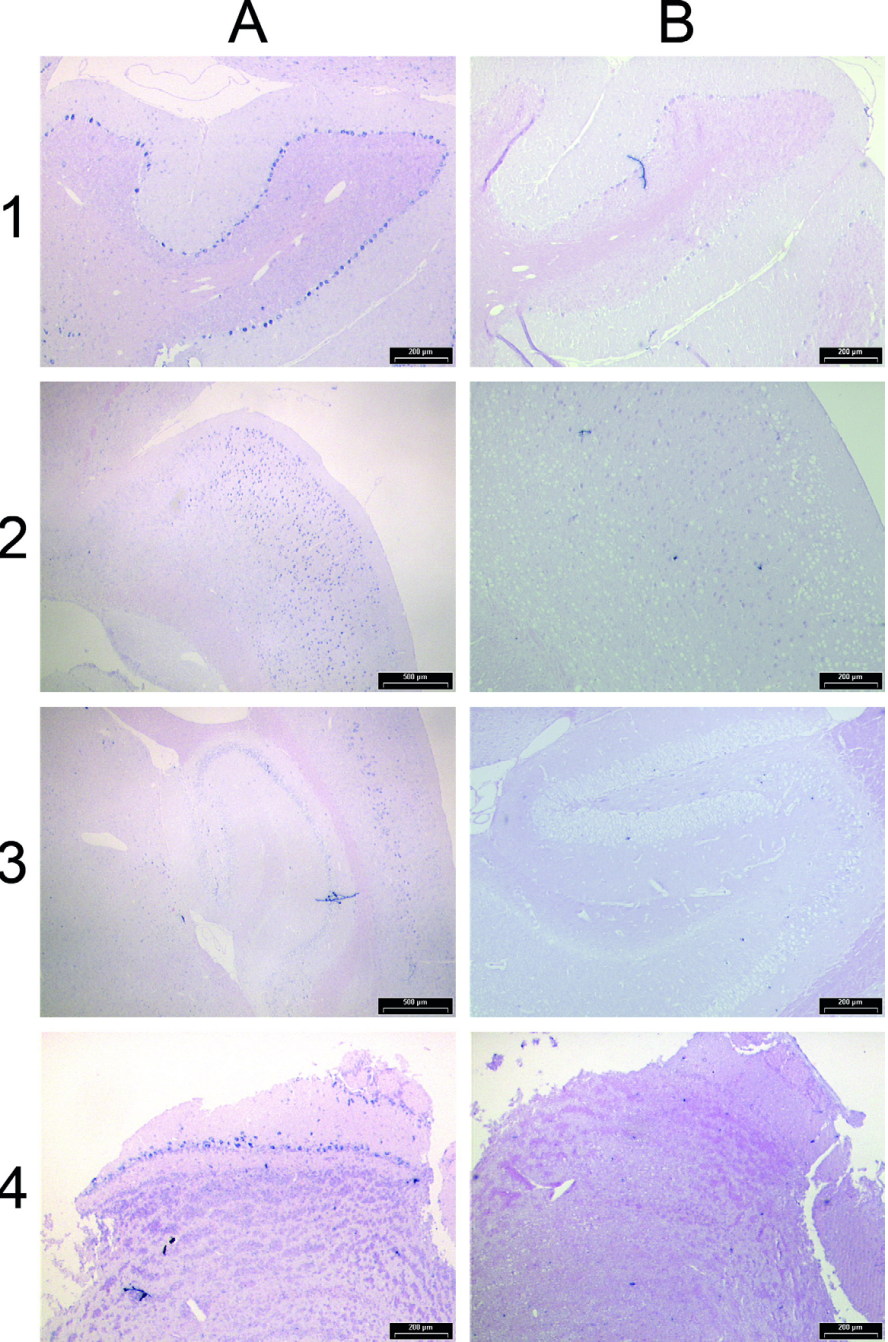
**RNA in situ hybridization of variant *Il4i1_2 *in different areas of adult mouse brain**. Shown are sagittal sections from cerebellum (1), cortex (2), hippocampus (3) and olfactory bulb (4). Probes were transcribed from the joining exons specific for variant *Il4i1_2*, located between the previously known *Nup62 *and *Il4i1 *gene loci. Signals (in blue) obtained with the antisense probe are on the left (A), and those obtained with the sense probe are on the right (B). The scale bar is 500 μm in 2A and 3A, and 200 μm in the other images.

Having identified expression of the *Il4i1_2 *variant in a tissue other than B lymphocytes, we next carried out RNA in situ hybridization to identify a possible cell-type specificity of this expression, and to find other tissues and cells where the variant is expressed. Expression of variant *Il4i1_2 *was found in testis to be predominantly in Sertoli cells at the periphery of the ducts (blue spots in Fig. 5, panels A1 and A2). In contrast to the Northern analysis, where brain did not have detectable expression of the *Il4i1_2 *variant, RNA in situ hybridization revealed expression of the variant transcript in the adult mouse brain (Fig. 6). Purkinje cells (cerebellum), cells of the hippocampus, and mitral cells in the olfactory bulb were specifically stained with the *Il4i1_2 *specific antisense probe (Fig. 6). Even though expression in some cell types within the brain was strong, overall expression of variant *Il4i1_2 *in the brain was weak, matching the results obtained with pooled brain tissue in the Northern analysis. No signals were detected in adult liver and kidney or in any of the embryonic stages by RNA in situ hybridization (not shown).

## Discussion

We here report a novel transcript variant of the *IL4I1 *gene, which is a product of two exons from the previously described *NUP62 *gene, two apparently joining exons mapping between the reported *NUP62 *and *IL4I1 *gene loci, and six exons of the known *IL4I1 *gene. Expression of that variant is driven by the assumed *NUP62 *gene promoter with high tissue and cell type specificity. The protein encoded by the variant *IL4I1_2 *transcript is essentially the same as that of the originally described Il4i1 protein [32], since the primary structures of the encoded proteins are identical after probable cleavage of the predicted signal peptides. Although a different functionality of the variant signal peptides cannot be excluded [40], the expression of this otherwise B-cell specific gene in testis and brain already adds significantly to the previously known properties of that gene and the encoded enzyme. The tissue specificity of the reported *IL4I1 *promoter [31] appears to be essential for survival, and expression of that gene appears to be tightly controlled. Given the function of the encoded protein, a LAAO enzyme, such restriction of protein expression makes sense. Limiting *IL4I1 *expression to B cells would take reference to the specific function of that cell type (e.g. antigen processing). In contrast, the Il4i1 protein is likely not involved in the immune system/antigen processing when expressed in testis or the brain. While the function of that protein in these tissues thus remains to be established, a possible involvement in disease should be analyzed. The lysyl oxidase (LOX) has been found at elevated levels in amyotrophic lateral sclerosis (ALS) and in superoxide dismutase (mSOD1) knockout mice (which exhibit an ALS-like syndrome) and is believed to be involved in the progression of ALS [41]. The LAAO activity of Il4i1 makes this protein a new candidate not only for ALS, but also for other diseases associated with the death of Purkinje cells [42]. For example, the chromosomal location of the *IL4I1 *gene at 19q13.31 has been described as candidate region for spinocerebellar ataxia type SCA19. Elevated expression levels of *IL4I1 *have also been reported in primary mediastinal large B-cell lymphoma [43], thus associating this gene with cancer as well. Further experimentation will be necessary to establish a possible role of the variant *IL4I1_2 *in any of these or other diseases.

The previously described *IL4I1 *promoter appears to be strictly specific for B-cell expression. It does not contain a CpG island and is reported to be induced for instance by STAT6 [31]. In contrast, the *IL4I1_2 *variant in the human is likely to be expressed exclusively in testis and brain. The *NUP62 *gene has a CpG island and is ubiquitously expressed. In consequence, pre-mRNAs are spliced to produce the novel variant only in testis and brain. However Purkinje and Sertoli cells also require functional nuclear pore complexes to survive. Correct amounts of both mRNAs need to be generated within the cells. The amounts could be regulated most likely at the splicing and/or polyadenylation levels, or by specific mRNA degradation. In consequence, the variant *IL4I1_2 *transcript is indicative of a so far undetected mechanism of gene regulation. While the presence of alternative promoters is a common theme in many genes, the cell-type specific expression of two genes from one promoter is novel, especially when the transcripts contain exons from both genes.

Thus far, gene fusions had mostly been associated with disease [44]; for example, trans-splicing is associated with viral infection [45]. However, the process reported here occurs in normal individuals and could be essential in the expressing cell types. Apparent joining of genes as indicated by cDNA sequences takes place at a rather high rate, but in many cases these cDNAs are likely to have been the result of errors in the pre-mRNA processing machinery [46]. One example is AK074097, which points to a fusion between *IL4I1 *and the downstream gene encoding TBC1 domain family member 17. However, these genes are oriented tail to tail, and the sequence structure of AK074097 is not supported by any further cDNA data. AK074097 even extends into the next further downstream gene AKT1S1. The "splice variant" represented by this cDNA therefore most likely originated from the lack of transcriptional termination and mis-splicing of cryptic "exons". This cDNA could thus be regarded as biological noise [46]. While being probably not of functional relevance, this and many other similar cDNA sequences (also IMAGE:5168029) raise questions as to the fidelity of RNA production and processing in cells, and as to the requirement of biological systems to be able to tolerate such events. Since errors at the RNA level are not inherited per se, the observed phenomena presumably are indicative of the flexibility and stability of the cellular system, rather than that these RNAs themselves would contribute to the evolutionary principle directly. Our findings now suggest that promiscuity of the pre-mRNA processing machinery is a required mechanism on a higher than previously reported [5,6,47,48], i.e., a trans-gene level, and that it is regulated at tissue and cell-type levels.

**Figure 7:**
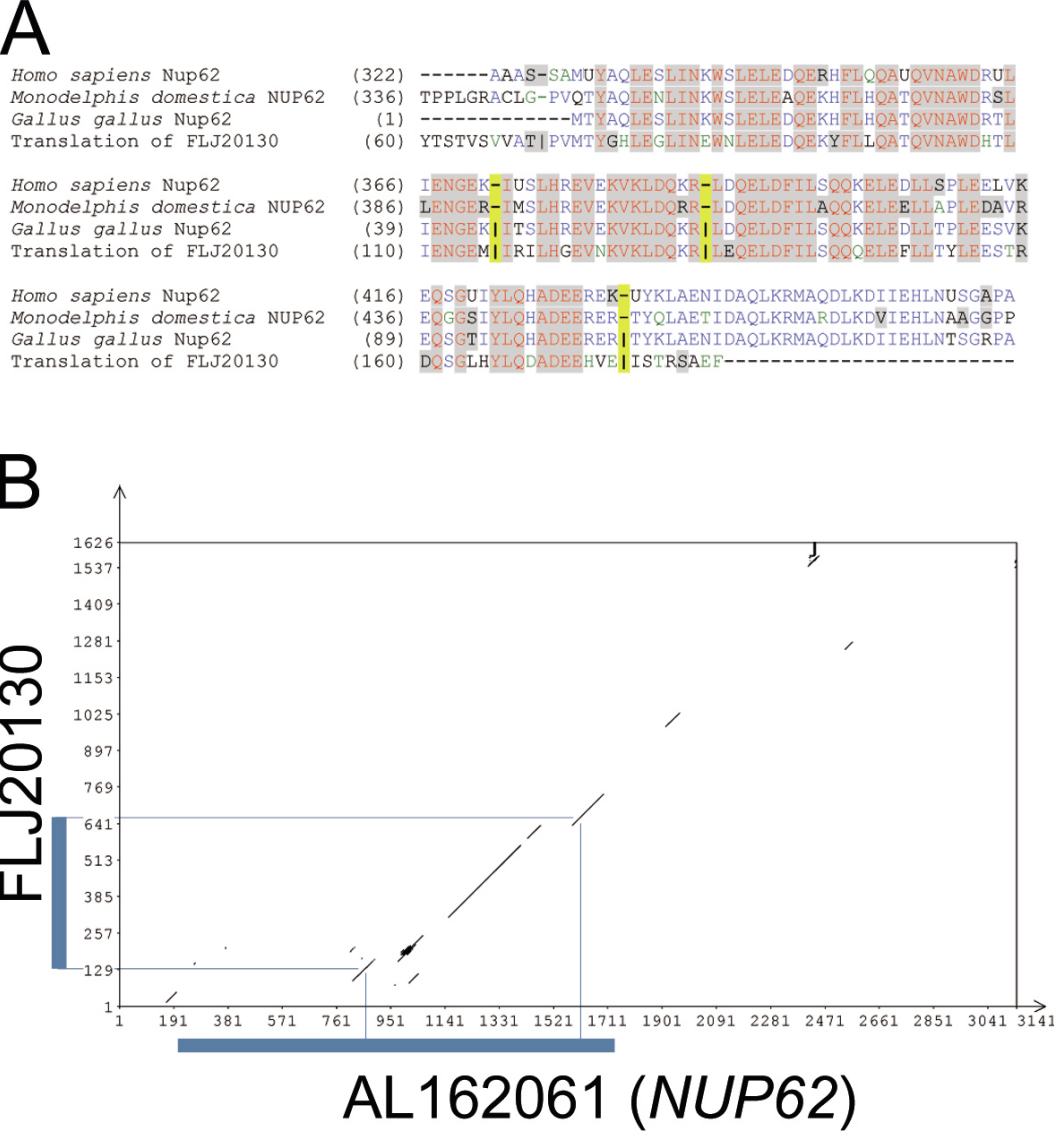
**Alignment of *NUP62 *and FLJ20130**. (A) Protein sequences of human, opossum and chicken Nup62 were aligned with the peptide encoded by cDNA FLJ20130. While most of the mammalian *NUP62 *genes do not contain introns, the chicken *NUP62 *gene and the human gene encoding FLJ20130 do. Exon boundaries in the latter two sequences are indicated with pipes (|), and the lack of introns is indicated at the respective positions by dashes (-). The positions of exon boundaries are additionally highlighted in yellow. The N terminus of chicken Nup62 is probably incomplete. (B) Dot-plot of human nucleotide sequences FLJ20130 (presumed ancestor of *NUP62*) and AL162061 (*NUP62*). Windowsize was 55 bp, and stringency was set to 60% sequence identity. Coding regions in the two cDNAs are indicated with blue bars, and ORF boundaries are linked to the dot-plot alignment. The alignment extends past the protein coding sequences. The terminal and coding exon of AL162061 starts at position bp 129 and comprises the assumed retroposon.

Several questions remain unanswered. Why and how is the pre-mRNA spliced to specifically produce the variant *IL4I1_2 *mRNA? Is transcription of RNA polymerase past the 3'-terminal exon of *NUP62*, which is required to join exons from the apparent *NUP62 *and *IL4I1 *genes, restricted to the cell types and tissues where the variant is detected, or is the tissue specificity of that variant mRNA determined in the splicing/polyadenylation process? Why is *IL4I1 *expression driven by the *NUP62 *promoter at all? More globally, is this a unique mechanism or are there more genes that are driven by the promoters of upstream genes? Are there other cases where an apparently leaky splicing mechanism could be favourable over the risk of erroneous transcription from a more promiscuous promoter? And finally, how did this mechanism evolve? The evolution of this mechanism would have required at least three events to happen, probably in early eutherian development: 1) the installation of neighborhood and orientation of these two genes, 2) the continuation of transcription beyond the *NUP62 *translated exon and its transcription termination signals, and 3) the development of tissue specificity for *NUP62 *and *IL4I1_2 *pre-mRNA processing. Selective pressure appears to have favoured preservation of the status quo. Sequence conservation of those exons that are specific for the variant is even higher than that of exons 1 and 2 of the previously reported *IL4I1 *transcript. This could hint to an essential function of the variant and to the possibility that it was not *IL4I1 *that integrated downstream of *NUP62*, but that instead *NUP62 *integrated into the *IL4I1 *gene. The latter hypothesis is supported by the fact that the complete *NUP62 *ORF is located on one exon in mammals, while it is split into several exons in other eukaryotes. Thus the so-called *NUP62 *promoter might actually be an ancient *IL4I1 *promoter that triggered expression of two independent ORFs after integration of a *NUP62 *retroposon. An immediate question would follow: what happened to the original *NUP62 *gene in eutherians? The cDNA FLJ20130, which maps to human chromosome Xq22.3 encodes a protein that is homologous to part of Nup62, namely the most conserved nucleoporin Nsp1-like C-terminal domain (IPR007758). That domain is fundamental for interaction with Nup82, another protein of the nuclear pore complex. The exon/intron structure of at least three exons in the FLJ20130 locus is the same as in the chicken *NUP62 *gene (Fig. 7A). The conservation of FLJ20130 and *NUP62 *extends into the 3'UTR of FLJ20130, which is however part of the coding region of *NUP62 *(Fig. 7A). In human, dog, mouse and opossum, the FLJ20130 gene is flanked by CXorf 41 (upstream) and FLJ11016 (downstream) and their orthologs, respectively. The same homologous genes flank the *NUP62 *gene in chicken in identical order and orientation. FLJ20130 in human Xq22.3 might consequently be a remnant of the ancient *NUP62 *in mammals, having lost a number of exons and much of its coding region. Other examples of retrogenes have been reported [49]. In contrast to the ubiquitous expression of *NUP62*, EST data from mouse and human suggest that expression of FLJ20130 is mostly in early development. These findings indicate this probable ancient form of mammalian *NUP62 *may still be expressed but is likely to have acquired a novel function. Presence of the Nsp1_c like region, however, could implicate this protein to be involved in the nuclear pore complex as well.

The mechanism that determines the processing of *NUP62/IL4I1_2 *pre-mRNAs into its final form also remains to be identified. An attractive model would be the frequently observed use of alternative polyadenylation sites that is coupled to alternative splicing [50]. Then the terminal exon of *NUP62 *could be interpreted as "merely" an alternative 3'-end of the *IL4I1 *gene, or the downstream exons of *IL4I1 *were alternative ends of the *NUP62 *gene. A possible *NUP62 *retroposon would have contained a polyadenylation signal and a polyA-tail. Consensus polyadenylation signals (AAUAAA) are present in the *NUP62 *gene and transcripts while the polyA tail appears to have vanished since the time of integration. The downstream sequence element needed to make the polyadenylation signal functional [51] and to terminate transcription must have been present within the intron of the *IL4I1 *gene into which the retroposon inserted.

## Conclusion

We have identified and verified a novel mechanism for regulation of gene expression that involves the transcription of two genes from the same promoter and the processing of two variant mRNAs from probably the same pre-mRNA. The encoded proteins are completely unrelated. Conservation of this mechanism in eutherians suggests both transcripts and the encoded proteins are essential for survival. Finally, our finding puts the current definition of the term "gene" in question, as the variants we have identified and analyzed are clearly the product of two genes. In addition to one promoter driving the expression of these genes, two of the formerly named *NUP62 *exons are also part of the *IL4I1_2 *variant. Should these exons be counted as belonging to the *NUP62 *or to the *IL4I1 *genes? One current definition of a gene is "a complete chromosomal segment responsible making a functional product" [52]. The chromosomal segment encoding the B-cell variant of *IL4I1 *appears completely separate from that of *NUP62 *and thus fulfils all criteria of the above definition. This is not true however for the newly detected *IL4I1_2 *variant. *NUP62 *and *IL4I1_2 *share noncoding regulatory DNA sequences, exons and introns within one chromosomal segment. The functional sequences of *NUP62 *and *IL4I1_2*, however, are unique and distinct, which is another criterion used to separate two genes. In consequence, the above definition of a "gene" should be put in question. Nature may have more surprises to reveal, and with increasing amounts of data on genomes, transcriptomes and proteomes being collected and analyzed, other paradigms may require revision.

## Methods

### Identification of splice variant

The cDNA IMAGE:4822638 (Acc:BC026103) was cloned and sequenced by the Mammalian Gene Collection [26]. More cDNAs were identified in the University of California, Santa Cruz (UCSC) genome browser [53,54] (assembly of May 2004), based on their EST sequences to cover part of the *IL4I1_2 *variant (IMAGE:5168029, IMAGE:5171014, IMAGE: 5742307, IMAGE:4838597). All these cDNAs were obtained from The German Resource Centre for Genome Research (RZPD; [55]) and completely sequenced with help of walking primers [56]. Sequences were assembled and aligned using the Staden package [57] to identify base substitutions and other alterations from the predicted consensus sequence.

### Comparative genomic analysis

Comparative genomic analysis of the *IL4I1_2 *variant was done with help of the UCSC genome browser [53], which indicated variant cDNAs from mouse [58] (ESTs Acc:BY100275, BY099330, BY087056, BY092834, BY088421) and rat (Acc:CV117152). Alignment of protein sequences was done with Vector NTI software (Invitrogen). Synteny of genomic regions downstream of the *NUP62 *orthologs was analyzed in the genome assemblies and datasets of human (hg17), chimpanzee (panTro1), dog (canFam1), mouse (mm5), rat (rn3), opossum (monDom1), chicken (galGal2), *Fugu *(fr1), and *Drosophila *(dm1), all in the UCSC genome browser [54].

### Northern hybridization

Multiple tissue Northern blots with poly-(A)+-RNA from mouse embryonic (Cat.# 636810) and mouse adult tissues (Cat.# 636808) were obtained from BD Biosciences Clontech. A probe specific for the mouse variant *Il4i1_2 *transcript was generated with the primers mmNupIlR1 (GAAGAACACAGGCAGATGCCCTG) and mmNupIlS1 (TGCATGGTGGTCTTTGTGGGGC), which were used to amplify the mouse joining exons 2 and 3 of the variant *Il4i1_2 *(equivalent to the human exons 3 and 4 indicated with red arrowheads in Fig. 1) from mouse testis RNA via RT-PCR. The 208 bp PCR product was cloned into the pCRII vector (Invitrogen), and sequence verified. Filters were hybridized with ^32^P-labelled purified PCR products from that clone. Hybridization was overnight in Church solution (1M Na_2_HPO_4_, 1M NaH_2_PO_4_·H_2_O, 10mM EDTA, pH8.0) at 65°C. Filters were washed once in 0.1% SDS/0.1xSSC for 10 min, once in 0.1% SDS/0.3xSSC for 10 min, and then exposed to Kodak Bio Max at -80°C.

### RNA in situ hybridization

RNA in situ hybridization was performed on embryo sections at stages 10.5, 12.5, 14.5, 16.5 and different tissues of adult mice (testis, kidney, liver and brain). Embryos were isolated from pregnant NMRI mice. The day of plug detection was considered to be day 0.5 post conception (dpc). The tissues and embryonic stages were fixed over night in 4% paraformaldehyde (PFA) in phosphate-buffered saline (PBS) at 4°C. The tissues from adult NMRI mice were isolated after perfundation with 4% PFA in PBS. After embedding in paraffin, 6 μm sagittal sections were mounted on Superfrost+ slides. Cloned PCR products (see Northern hybridization) were sequence verified to identify orientation of the product within the vector. Antisense (T7) and sense (SP6) riboprobes labeled with digoxigenin-UTP (Enzo) were generated by in vitro transcription (Roche), after linearization of the constructs. Pre-treatment, hybridization and washing were carried out using a Ventana discovery system. Sense or antisense RNA probes were hybridized at 100ng RNA/ml in hybridization buffer in a volume of 100 μl/slide. Slides were analyzed using a Leica microscope.

Photographs were taken with a liquid crystal display (LCD) – camera (Power head, Sony) using AnalySIS software (Soft imaging System GmbH). The figures were assembled using Adobe Photoshop.

## Authors' contributions

SW designed the study, carried out the sequence analysis and drafted the manuscript. AKK carried out the experimental research and helped to draft the manuscript. AP participated in study design and coordination. All authors read and approved the final manuscript.
